# Decreased Production of TNF-α and IL-6 Inflammatory Cytokines in Non-Pregnant Idiopathic RPL Women Immunomodulatory Effect of Sildenafil Citrate on the Cellular Response of Idiopathic RPL Women

**DOI:** 10.3390/jcm10143115

**Published:** 2021-07-15

**Authors:** Monika Kniotek, Michał Zych, Aleksander Roszczyk, Monika Szafarowska, Małgorzata Maria Jerzak

**Affiliations:** 1 Department of Clinical Immunology, Transplantation Institute, Medical University of Warsaw, Nowogrodzka 59, 02-006 Warsaw, Poland; michal.zych@wum.edu.pl (M.Z.); aleksander.roszczyk@wum.edu.pl (A.R.); 2 Department of Gynecology and Oncological Gynecology, Military Institute of Health Sciences, Szaserów 128, 04-141 Warsaw, Poland; monika.szafarowska@wp.pl (M.S.); mmjerzak@wp.pl (M.M.J.); 3 Private Practice Małgorzata Jerzak Ostrołęcka 3 D, 05-510 Konstancin-Jeziorna, Poland

**Keywords:** cytokines, recurrent pregnancy loss, sildenafil citrate, Treg cells, Th1/Th2 cells, Th17 cells

## Abstract

Sildenafil citrate (SC), a PDE5 inhibitor, a drug for erectile dysfunction (ED) and pulmonary hypertension (PAH), was found to exert a positive effect on pregnancy outcomes when administered intravaginally before conception. In our previous studies, sildenafil increased endometrial thickness and significantly decreased peripheral blood NK cell activity after the intravaginal administration in women with recurrent pregnancy loss (RPL). No data are available to confirm the effect of sildenafil on maternal T cell populations involved in shaping fetal-maternal tolerance and NK cell activity. Thus, the present study aimed to establish if SC influences NKT cells or the axis of Th17/Treg cells and Th1/Th2 cytokine production. Materials and methods: Twenty-one healthy fertile women and twenty-two nonpregnant women with idiopathic RPL were studied. The ELISA method was used to evaluate the production of cytokines, including IL-2, IL-12p40, IL-4, IL-10, IL-6, IL-17, IL-21, TGF-β, TNF-α, and IFN-γ in PBMC culture supernatants before and after supplementation with the physiological concentration of SC. The percentages of NKT (CD56^+^CD3^+^CD44^+^CD161^+^), Treg (CD4^+^CD25^+^FOXP3^+^) and Th17 (CD4^+^CD25^+^IL-17A^+^) cells were determined with flow cytometry method. Results: Unexpectedly, we found that the PBMCs of patients with RPL produced a significantly lower level of inflammatory cytokines (TNF-α and IL-6) and a higher level of anti-inflammatory cytokines (TGF-β and IL-10). SC significantly decreased IL-6, IL-12 and increased TGF-β cytokine concentration in fertile women. In the case of RPL patients’ PBMCs, SC improved the production of TNF-α and IL-10. Conclusions: Lower concentration of proinflammatory cytokines in idiopathic RPL women compared to fertile women might suggest the exhaustion of the immune system. The emphasized production of IL-10 by SC partially explains the previously observed downregulation of NK cell activity in RPL patients. The immunomodulatory effect of the drug might be utilized in anti-inflammatory therapies and help achieve positive pregnancy outcomes in women with reproductive failure due to a Th1/Th2 imbalance.

## 1. Introduction

A positive pregnancy outcome is strictly dependent on maternal immune system tolerance. Several immune mechanisms involving both innate and adaptive immune responses are engaged at the fetal-maternal interface [1]. Conventionally, recurrent pregnancy loss (RPL) was defined as three consecutive losses earlier than 20 weeks of gestation. However, testing women after two losses could spare them another pregnancy failure. Therefore, the definition was modified by lowering the number of spontaneous losses to two [2]. Unexplained RPL is a growing health problem worldwide. It is estimated that RPL affects more than 1% of the general population of pregnant women, and only half of RPL cases could be explained after medical investigation [2,3]. Each miscarriage increases the risk of the next miscarriage to 15% [2].

Recently, it has been shown that 95% of lost embryos have a normal karyotype and that the alloimmune rejection-type activity of maternal humoral/cellular immunity or high NK activity accounts for a great proportion of RPLs [4]. The maternal immune system prepares the uterus for the embryo, which from the immunological point of view, is a kind of haploidentical allograft. Immune cells found at the implantation site include the subpopulations of decidual Natural Killer cells (dNK), T cells (Th1, Th2, Th17 and Treg), Natural Killer T cells (NKT), ‘educated’ macrophages and dendritic cells [1]. Decidual NK cells (dNK cells), an NK2 type cell producing IL-4, Il-5 and IL-13 cytokines, constitute 70% of immunocompetent cells in the endometrium [5]. They normally control angiogenesis and the implantation process (the depth of trophoblast invasion). The dysregulation of dNK cell activity may lead to the termination of pregnancy, preeclampsia or gestational trophoblastic disease [5,6,7]. The specialized regulatory T cell (Treg) population (mostly paternal antigen-specific Treg cells) by releasing a large amount of TGF-β, IL-10 and IL-35 an inhibitory cytokine, is pivotal for dNK cell inactivation and maintaining immune tolerance to paternal antigens [5,8]. The overexpression of pro-inflammatory cytokines like IL-1β and IL-6 blocks the development of Treg cells and induces the differentiation of Th17 cells in feto-maternal interphase [4,9,10,11]. IL-17 was found in the cyto- and syncytiotrophoblast whose overexpression may lead to the activation of immune response, including NK cell cytotoxicity towards the trophoblast [12]. Normally, IL-17, along with IFN-γ and TNF-α cytokines, play a major role in angiogenesis in the uterus [4,9,10,11], but the chronic exposure to Th1 type cytokines leads to the enhancement of dNK cell cytotoxicity activation [13].

Our previous research showed that sildenafil citrate (SC) suppositories improved endometrial thickness and uterine blood flow. The drug was able to modulate maternal immunity by decreasing NK cell activity. This effect on NK cells was shown in in vivo and in vitro studies [14]. Sildenafil, through an indirect increase in nitric oxide (NO) production, might influence the methylation pathway in the immune cells [15]. SC increases cellular cGMP levels through competition for the phosphodiesterase binding site with cGMP, thus inhibiting the degradation of cGMP to GMP [15]. A high level of cGMP results in increased NO production, and consequently causes the relaxation of vascular smooth muscles, increases vasodilation and modulates immune response [15,16]. Therefore, sildenafil citrate is currently applied for the treatment of such intragestational complications as intrauterine growth restriction (IUGR) [6,17,18,19], low birth weight [5], preeclampsia or idiopathic recurrent pregnancy loss (RPL) [5,7,19,20,21,22]. Some experiments conducted on animal models, where SC was orally administered, suggested that SC might decrease IL-1, and TNF-α in the placenta of pregnant mice [23,24]. Some authors reported a gender-specific action of sildenafil on immune cells, where SC diminished IL-2 and IL-6 production in the serum of female mice [25]. Contradictory results obtained from female and male mice and in vitro cultures indicated the gender- and tissue-specific action of sildenafil [25]. Additionally, Pifarré et al. showed that SC might upregulate Treg cell percentage and Grb1 protein expression in cultured splenocytes [26].

Taking the above into account, the modulation of NK cell activity in RPL women after SC treatment might be achieved through cytokine production modulation. Therefore, our recent study focused on sildenafil-mediated effects on the subpopulations of Treg/Th17 and Th1/Th2/Th17 cytokine production, studied in the cultures of the PBMC of healthy fertile women and women with idiopathic RPL.

## 2. Material and Methods

The study was approved by the Bioethics Committee of the Medical University of Warsaw (No. KB/192/2015). All measurements, interventions and blood collections were performed after informed consent had been obtained from each woman participating in the study using the bioethics committee-approved protocol. Each of the participants was informed about the purpose of this study.

### 2.1. Control Group

The subjects enrolled in this study were volunteer participants. They were recruited from the Department of Gynecology and Gynecologic Oncology, Military Institute of Health Sciences, Warsaw, Poland. The control group consisted of 21 fertile women without disorders in the obstetric-gynecological and internal medicine history. None of the subjects included in the control group reported any problems regarding conception; all subjects declared a normal course of pregnancy and delivery. Besides, none of the control subjects was treated for any internal disorders. Women on oral hormonal contraception or other forms of hormonal treatment and women with hormonal intrauterine devices were excluded from the study. Transvaginal ultrasound scans were performed in all patients between days 3 and 5 of the menstrual cycle to reveal the normal morphology of the uterus, endometrium and appendages.

The blood collected from the women between 16–25 days of the menstrual cycle was vested in heparin tubes.

### 2.2. Study Group

The subjects enrolled in this study were volunteer participants. They were recruited from the Mediva Medical Center in Warsaw between February 2016 and May 2017. One hundred and fifty patients with RPL were evaluated. However, 22 patients (aged 36.70 ± 4.48) with unexplained RPL were finally included in the study group. Recurrent pregnancy loss was defined according to the American Society for Reproductive Medicine (ASRM) guideline as two or more consecutive spontaneous miscarriages before the 20th week of gestation [23,24]. Complete medical, surgical and social histories were obtained in all cases. All the women with a history of RPL were investigated in terms of any identifiable causes of abortion. The patients included in the study presented no anatomic, genetic, microbiological, immunological or hormonal causes of abortions. Transvaginal ultrasound, hysterosalpingography or hysteroscopy did not reveal any abnormalities in the patients’ uteri. Peripheral blood chromosome assessment confirmed normal karyotypes. All laboratory tests including hormonal assessment revealed no abnormalities. Besides, none of the subjects was treated for any internal disorders or had surgical interventions. In our study, we included 11 women who met the PCOS criteria (the Rotterdam ones) and 18 women with an MTHFR mutation who presented no abnormalities in laboratory tests. All the women were treated according to guidelines and previous publications. Despite the optimized treatment, they experienced another obstetric failure [23]. Table 1 showed the characteristics of the study and control group. According to our study protocol, blood samples were collected from the study group RPL patients six months after the last miscarriage, so the immunological status of the patients had been normalized before the research. The blood was collected from the patients between days 16–25 of the menstrual cycle, as in our previous study [14].

### 2.3. Cell Preparation

Fasting peripheral blood samples of fertile and RPL women were vested into heparin tubes in the morning. The isolation of peripheral blood mononuclear cells (PBMCs) and cell culture procedures were carried out as described in our previous study, which revealed that sildenafil dramatically decreased NK activity [14]. Concisely, PBMCs were isolated from the peripheral blood of 22 women with RPL and 21 fertile women by histopaque gradient centrifugation (Histopaque density 1.077 g/mL, MERK, KGaA, Darmstadt, Germany), as previously described [14]. Next, the cells were washed twice in PBS without Ca^++^ and Mg^++^ ions, (PBS, Corning, NY, USA) and resuspended in complete culture medium (RPMI medium, Corning, NY, USA) supplemented with 10% heat-inactivated fetal calf serum (FCS, MERK, KGaA, Darmstadt, Germany), 2 mM L-glutamine (MERK, KGaA, Darmstadt, Germany), 100 µg/mL Penicillin-Streptomycin (Gibco, Thermo Fisher Scientific, Waltham, MA, USA); 0.1 mM/mL HEPES potassium salt (MERK, KGaA, Darmstadt, Germany).

#### Sildenafil Concentration in Cultures

The concentration of sildenafil citrate after the oral administration of 200 mg in humans reaches 250 ng/mL after 0.5–2.5 h [27] in the serum, but 96% of the drug is protein-bound, so the free active concentration of sildenafil in the serum is only 4% of the total level [28]. The intravaginal administration of suppositories with sildenafil citrate results in a 40-fold increase in C max of the drug in the uterus compared to oral administration [29]. Therefore, the concentration of the drug used in the study was 400 ng/mL (0.6 µM) in the cell culture.

### 2.4. Cell Cultures

#### Cytokine Determination

Cells were cultured in 24-well plates at the concentration of 1 × 10^6^/mL (NUNC 24-well plates, Thermo Fisher, Scientific) in two versions: PBMC in medium and PBMC in medium supplemented with 400 ng/mL sildenafil citrate (MERK, KGaA, Darmstadt, Germany). After 48 h of incubation at 37 °C in a humidified atmosphere containing 5% CO_2,_ the cells were centrifuged at 1800 rpm for 10 min. The cells were collected for Treg/Th17 determination and the supernatants were stored at −80 °C for the ELISA tests. According to the instruction included in Human Th17/Treg phenotyping KIT (BD Pharmingen, San Diego, CA, USA we also performed cultures with cells stimulated with phorbol-ester (PMA) (MERK, KGaA, Darmstadt, Germany)) and ionomycin (MERK, KGaA, Darmstadt, Germany), but PMA dramatically decreased FOXP3 expressing cells, so we disregarded those results in further analyses.

The concentrations of selected cytokines (IL-2, IL-12p40, TNF-α, INF-γ IL-17A, IL-21, IL-10, IL-4, IL-6, TGF-β) in culture supernatants were measured with the double-antibody sandwich enzyme-linked immunosorbent assay (ELISA), according to the manufacturer’s instructions to determine the level of cytokines. The concentrations of cytokines were calculated from the standard curve of linear regression according to the manufacturer’s instruction (ELISA-kits, Sun Red, Biotechnology Company Co. Ltd., Shanghai, China). The levels of sensitivity of ELISA-kits were: IL-2—0.753 pg/mL, IL-12 (p40)—0.225 ng/mL, TNF-α—2.827 ng/mL, IFN-γ—1.706 pg/mL, IL-17—2.013 pg/mL, IL-21—4.723 pg/mL, IL-4—4.116 pg/mL, IL-10—1.142 pg/mL, IL-6—2.112 pg/mL, TGF-β—4 pg/mL, with the Intra-Assay CV < 10% and Inter-Assay: CV < 12%.

### 2.5. Flow Cytometry Analysis

#### 2.5.1. NKT Cells Immunophenotyping

To determine proinflammatory CD3^+^CD56^+^CD44^+^ CD161^+^ NKT cells, 1 × 10^6^ of cultured PBMCs with and without SC, were washed in PBS with 1% NaN_3_ and stained with anti-human antibody cocktails: CD3-PerCP (SK7 clone), CD56-Pe-Cy7 (B159 clone), CD44-APC (G44-26 clone), CD161-PE (DX12 clone) and CD3-PerCP, CD56-Pe-Cy7, IgG2b-APC (isotype control for CD44), IgG1-PE (isotype control for CD161) (all Antibodies, from Becton Dickinson, Franklin Lakes, NJ, USA), for 20 min in the dark. The same time single-color compensation control was performed, where 1 × 10^6^ PBMCs per sample were stained with single a.m. antibodies. After incubation, the cells were washed twice in 0.01% NaN_3_ PBS (Corning, NY, USA) for 5 min at 2000 rpm_,_ to discard the excess of unbound antibodies, and then they were suspended in 500 µL of FACS Flow Buffer (BD Pharmingen, San Diego, CA, USA [30,31]. A total of 1000 cells of unstained cells and compensation controls were acquired with FACS Canto II (Becton Dickinson, Franklin Lakes, NJ, USA), and compensation was calculated with 6.1.3 Diva software (Becton Dickinson, Franklin Lakes, NJ, USA). Then, at least 20,000 cell readouts of CD3^+^ CD56^+^ cells of each studied sample were acquired with a flow cytometer BD FACS Canto II and analyzed with 6.1.3 Diva software. The threshold for positive staining was determined using unstained cells for CD3- and CD56-positive cells and isotype controls were used for bordering CD3CD56CD161– or CD3CD56CD44–positive cells. Subsequently, the percentage of CD161 and CD44–positive cells was determined among CD3CD56 cells (Figure S1 in Supplementary Materials). Instrument performance was verified daily using the Cytometer Setup & Tracking (CS&T) system (Becton Dickinson, Franklin Lakes, NJ, USA), applying CS&T application settings to ensure comparable flow cytometry results over time.

#### 2.5.2. CD4^+^CD25^+^IL-17^+^, CD4^+^CD25^+^FOXP3^+^, and CD4^+^CD25^+^FOXP3^+^IL-17^+^ Cell Determination

The above-mentioned cells obtained from cultures were used for the evaluation of regulatory T lymphocytes (FOXP3-expressing CD4CD25 cells) and lymphocytes T producing IL-17 cells with a flow cytometry method according to the manufacturer’s instructions (Human Th17/Treg phenotyping KIT, BD Pharmingen, San Diego, CA, USA). Briefly, the cells were washed in Stain Buffer (1% FBS in PBS) and 0.5 × 10^6^ of cells were labeled with anti-human CD4-PerCP and anti-human CD25 FITC antibody (clone 2A3, BD Pharmingen, San Diego, CA, USA). After extracellular staining, the cells were fixed in Human FOXP3 Buffer A (BD Pharmingen, San Diego, CA, USA) for 20 min at room temperature (RT) in the dark, then washed and permeabilized with Human FOXP3 buffer C (BD, Pharmingen, USA). The cocktail of antibodies: CD4-PerCP/IL-17-PE/FOXP3-Alexa Fluor^®^ 647 was added to stain Th17 and Treg cells and the cells were incubated for 40 min in the dark at RT. After incubation, the cells were washed in Stain Buffer (BD, Pharmingen, San Diego, CA, USA) and suspended in 500 µL of FACS Flow (Becton Dickinson, Franklin Lakes, NJ, USA). At least 40,000 events of CD4^+^ CD25^+^ cells were acquired with a flow cytometer FACS Canto II, equipped with a 488-nm laser, a 633-nm laser and a 405-nm laser). The percentage of FOXP3, FOXP3/IL-17A, and IL-17A-positive cells was determined among CD4^+^ CD25^+^. Unstained cells were used as FMO control for CD4-positive cells and for CD25-positive cells. Then, among the gated CD4CD25-positive cells we determined CD4CD25IL-17, CD4CD25FOXP3, or CD4CD25IL-17FOXP3-positive cells (Figure S2 in Supplementary Materials). The results were calculated with BD FACS Diva 6.1.3. software.

### 2.6. Statistical Analysis

All statistical analyses were performed with Graph Pad Prism 8.00. A chi-square test was used for nominal variables. An odds ratio and 95% confidence interval were calculated for risk estimation.

The normal distribution of data was determined with the Shapiro-Wilk test. In order to determine the statistical significance between the control and study group samples, the unpaired *t*-test was used in case of the normal distribution of data, and the Mann-Whitney U test was used in case of non-normal distribution. The analyses of data inside the groups (samples after culturing with SC) were performed with the Wilcoxon signed-rank test in case of non-normal distribution, and the paired *t*-test for the normal distribution of samples. The *p* values below 0.05 (*p* < 0.05) were considered statistically significant. The trend was recognized as *p*~0.07. The data were shown as the median and interquartile range (IQR) in the figures.

The Pearson’s test was used to calculate the “r” factor and the significance of the correlation between the concentration of cytokines between the groups and the results were corrected for non-normal distribution of data with the Spearman’s rank test.

## 3. Results

No significant difference was observed between women from the control group and the study group regarding the age. The patients were tested for an MTHFR mutation, PCOS or IR. However, their status had been normalized with diet or folic acid supplementation before the research. Thus, we did not notice any correlation between MTHFR mutation, homocysteine level, IR or PCOS occurrence and the level of cytokines.

### 3.1. Secretion of Cytokines

**Figure 1:**
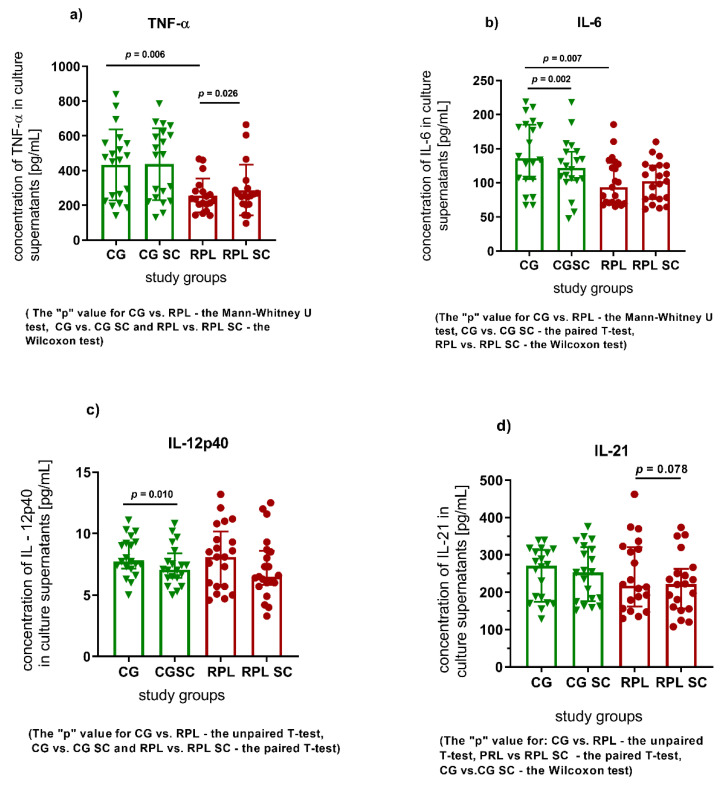
Changes in the concentration of pro-inflammatory cytokines in the culture supernatants of the PBMC cells of fertile and RPL women supplemented with 400 ng/mL sildenafil citrate, (**a**) TNF – α concentration in culture supernatants, (**b**) IL-6 concentration in culture supernatants, (**c**) IL-12p40 concentration in culture supernatants, (**d**) IL-21 concentration in culture supernatants, (CG—control group, green symbols, *n* = 20; RPL—study group, red dots; *n* = 21; SC—sildenafil citrate; data shown as individual values, the median and IQR). The tests used to establish significant differences are given below graphs.

Surprisingly, the basic levels of TNF-α, IL-6 pro-inflammatory cytokines were significantly lower and the concentration of TGF-β was higher in RPL patients compared to healthy women (Figure 1a,b and Figure 3c, and Table S1 in Supplementary Materials).

**Figure 2:**
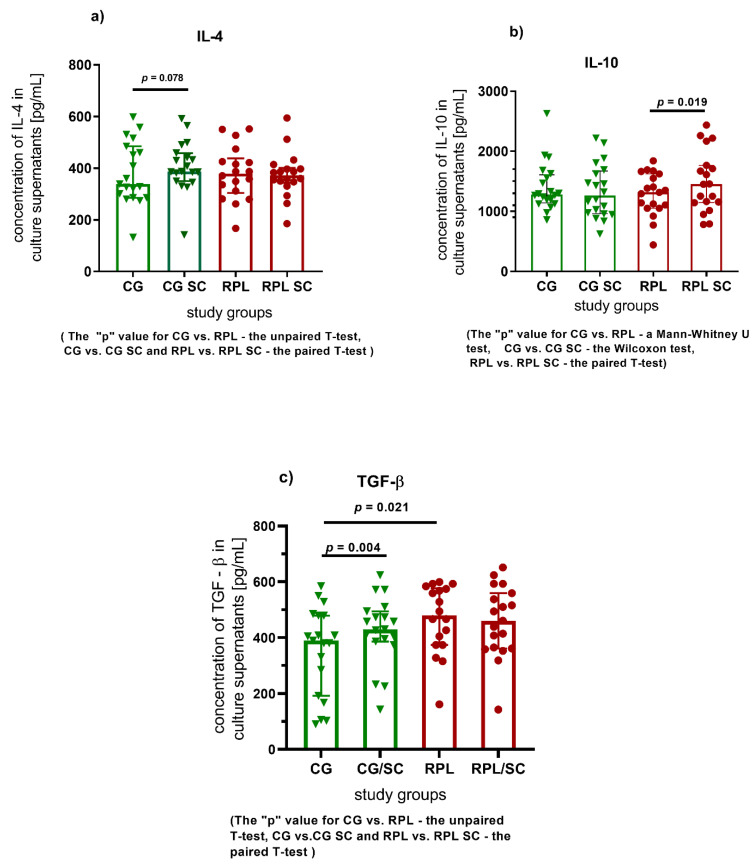
The comparison of anti-inflammatory cytokine concentrations in fertile and RPL women PBMC cultures, and the influence of SC on cytokine concentrations, (**a**) IL-4 concentration in culture supernatants, (**b**) IL-10 concentration in culture supernatants, (**c**) TGF – β concentration in culture supernatants, (CG—control group, green symbols, *n* = 20; RPL—study group, red dots; *n* = 21; SC—sildenafil citrate; data shown as individual values, the median and IQR). The statistical tests used to establish significant differences are given in the below graphs.

Considering the effect of SC on the production of selected cytokines by the PBMC of the fertile woman, SC significantly decreased the concentrations of IL-6 and IL-12, and im-proved TGF-β production. (Figure 1b,c and Figure 2c and Table S1 in Supplementary Materials). As regards the cultures of RPL patients, SC significantly enhanced the concentration of TNF-α and IL-10 cytokines (Figure 1a and Figure 2b and Table S1 in Supplementary Materials).

**Figure 3:**
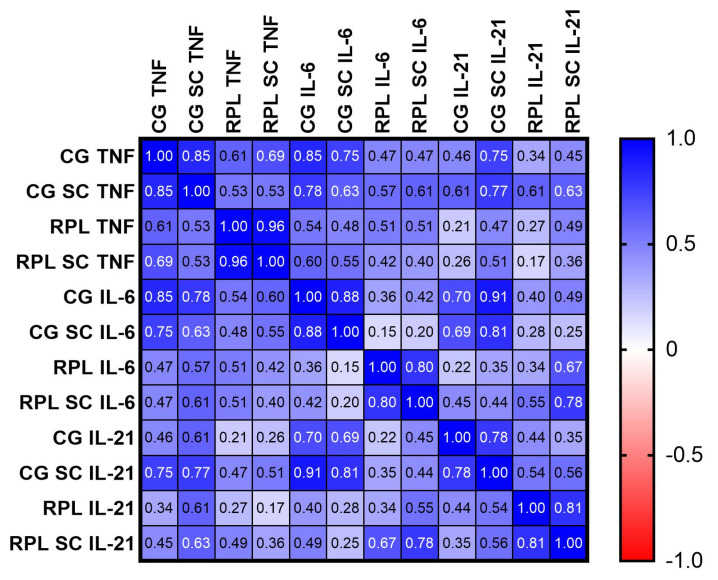
Pearson’s “r” factor correlations between inflammatory cytokines. Blue colors stand for a positive correlation r > 0, red colors r < 0. The graphs showing the correlation between particular cytokines: IL6 vs. TNF, IL-21 vs. TNF, IL-6 vs. IL-21 in CG and idiopathic RPL are shown in Supplementary Data, (CG—control group, *n* = 20; RPL—study group, *n* = 21; SC—sildenafil citrate).

The Pearson’s test was used to calculate the significance of the correlation between the concentration of cytokines between studied groups (Figure 3), and the results were corrected for non-normal distribution of data with the Spearman’s rank test (Figure S3). In fertile women, the production of the following cytokines was positively correlated: TNF-α vs. IL-6 (*p* = 0.0033), IL-21 vs. IL-6 (*p* = 0.0021), TNF-α vs. IL-21 (*p* = 0.0003), (Figure 3, Figures S3a,c,e and S4 in Supplementary Materials). The correlations were sustained for TNF-α vs. IL-6 (*p* = 0.028), TNF-α vs. IL-21 (*p* = 0.0003), and improved in the case of IL-21 vs.IL-6. (*p* = 0.00021) after SC addition in the control group (Figure 2 and Figure S3b,d in Supplementary Materials). In case of RPL patients, only the IL-6 vs. IL-21 connection reached statistical significance (*p* = 0.0001). (Figure 2 and Figure S3g in Supplementary Materials). We did not observe any differences of IFN-γ, IL-2, IL-17 or IL-4 concentrations between and inside the studied groups (Table S1 in Supplementary Materials).

**Figure 4:**
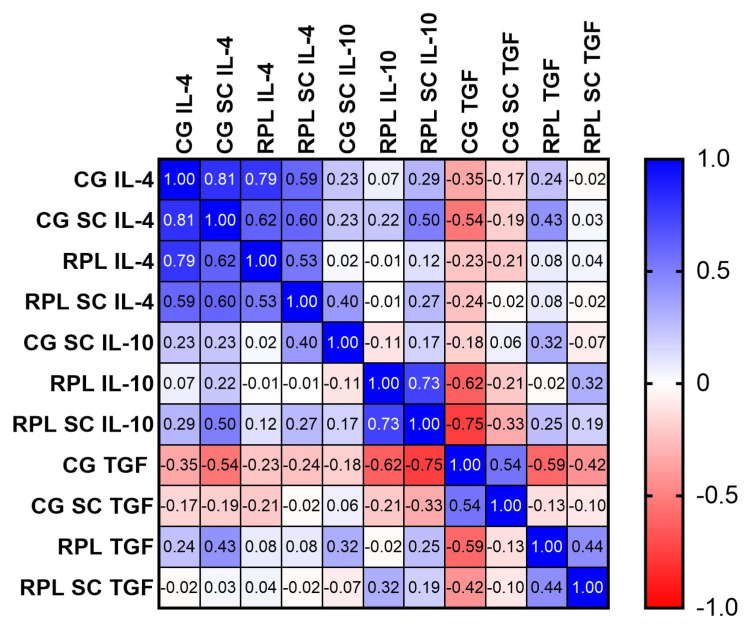
Pearson’s “r” factor correlations between anti-inflammatory cytokines. Blue colors stand for a positive correlation r > 0, red colors r < 0. (CG—control group, *n* = 20; RPL—study group, *n* = 21; SC—sildenafil citrate).

In the case of anti-inflammatory cytokines we notice a positive correlation only for IL-4 before and after treatment of SC in both studied groups and in RPL group for IL-10 after supplementation with SC (Figure 4).

### 3.2. The Effect of Sildenafil on Th17, Th17/Treg, Treg, and NKT Cells in PBMC Cultures

There was no statistical difference in the expression of FOXP3 in CD4+CD25^+^ cells of RPL patients compared to healthy women (Table 2). No significant correlation was observed between the concentration of cytokines and the expression of Treg or Treg/Th17 cells in the PBMC cultures of both studied groups.

Sildenafil had no significant effect on the percentage of CD4^+^CD25^+^FOXP3^+^ or CD4^+^CD25^+^FOXP3^+^IL-17^+^ cells in both studied groups (Table 2).

NKT cells was similar in both studied groups. The percentage of CD3^+^CD56^+^CD44^+^CD161^+^ cells remained almost unaffected by SC (Table 3).

## 4. Discussion

Most of our RPL patients had an MTHFR mutation and insulin resistance (IS), or PCOS. However, we did not observe any correlation between MTHFR mutation C677T and homocysteine level, PCOS or IR and PCOS, as reported previously [32]. Patients with MTHFR mutations did not present increased homocysteine levels, which was previously ascribed to increased pro-inflammatory cytokine concentration [33,34]. Moreover, the basic concentrations of TNF-α, IL-6 (Th1 type cytokines) were significantly lower in the RPL group of patients compared to fertile women. Furthermore, the concentration of TGF-β was increased in the study group. It has been believed until now that the exacerbation of Th1 response causes and precedes miscarriage [35,36]. Previous reports suggested a “Th2 bias” of the immune response in a successful pregnancy, but some yielded null findings, while others observed the protective effect of certain Th1 cytokines in human studies [37]. Nonetheless, we tested supernatants after 48 h of culturing PBMC, and not the patients’ sera at the time of miscarriage. Findings by Zanganeh et al. [38], Whitcomb et al. [37] and Yamada et al. [35] confirmed that an increased Th1 response was not found before conception, but only during the process of miscarriage [35,37,38]. Whitcomb tested the serum of women 10 days before miscarriage, and found low levels of several pro-inflammatory cytokines, IL-1β, IL-4, IL-6, IFN-γ and TNF- α, which confirms our findings [35,37]. Bates et al. [36] showed that the supernatants of the PBMC cultures of RPL patients stimulated with phytohemagglutinin (PHA) had a lower concentration of TNF-α and IFN-γ, and a higher concentration of IL-10 compared to the PBMC of pregnant women undergoing elective termination of pregnancy in the first trimester, concerning IL-10, it is in line with our outcomes. Yamada et al. [35] obtained similar results in the serum of women at 6–7 weeks of pregnancy with normal and abnormal karyotypes [35,36]. The authors suggested that a widely observed cytokine shift towards the Th1 phenotype might be the result and not the cause of miscarriage. Additionally, Aljamejl et al. observed a low concentration of IL-6 in the serum of RPL women, which confirms our results [39].

We noted a correlation of IL-6 and IL-21 cytokines after SC addition in RPL PBMC cultures only, as opposed to the control group where the correlations between the concentration of IL-6, TNF-α, IL-21 and IFN-γ vs. IL-17 cytokines were significant before and after treatment with SC. IL-6 and TNF-α are both controlled by MAPK kinases and IL-6 may positively influence IL-21 production, which may explain our observations. Additionally, IFN-γ was found to reciprocally influence the IL-17 release [40].

According to Salam et al., TNF-α regulates the trophoblastic metalloproteinases MMP-2, MMP3 and MMP-9, which enable trophoblast invasion. A higher TNF–α level was associated with a higher pregnancy rate, and it was established that the determination of TNF-α concentration might be a useful tool to predict embryo implantation [41]. Similarly, our RPL patients had a lower level of TNF-α than fertile women, which might be related to the incorrect implantation process [41].

Additionally, similarly to Ozan et al. [42], we founded a higher concentration of TGF-β in the case of RPL patients. Ogasawara et al. highlighted those women with severe recurrent miscarriages had an extremely elevated level of plasma TGF-β compared to the control value [43]. Moreover, Zhu et al. reported that the PBMC cells of RPL patients cultured with medium only had the highest spontaneous production of TGF-β among all studied groups of females [44]. Our results strengthened those findings and the suggestion that the elevated concentration of TGF-β might be a risk factor for recurrent pregnancy loss. The enhancement of TGF-β production might inhibit trophoblast invasion and result in a subsequent miscarriage [35,37,45].

TGF-β (in concert with other factors) promotes the development of peripheral Treg (pTreg), Th17 and Th9 cells. TGF-β signaling is dispensable for the induction of Foxp3 expression in Treg cells [46]. We did not find a correlation between TGF—β concentration with the expression of FOXP3 in Treg cells or Th17 cells percentage in our research. Furthermore, in the opposition to the previous findings [47], the difference in the expression of CD4^+^CD25^+^FOXP3^+^ or CD4^+^CD25^+^IL-17^+^ cells, in fertile and RPL women was insignificant.

The subsequent tested regulatory population was NKT cells. NKT cells rapidly secrete massive amounts of cytokines, including IL-1, IL-4, IL-5, IL-10, IL-12, IL-13, GM-CSF, IFN-γ and TNF-α, which consequently switch Th1, Th2, Th17 or Treg responses. Therefore, the excessive activation of NKT cells might extinguish pregnancy. However, the difference in the percentage of CD3^+^CD56^+^CD44^+^CD161^+^ (NKT) cells after 48 h of culturing of PBMC fell short of significance, although with a trend to increase in RPL patients [44,48,49].

Since our study involved the analyses of the PBMC production of cytokines isolated from the peripheral blood of women at least six months after their last miscarriage, it is impossible to comment on the local production of cytokines, percentage of Treg or NKT cells, e.g., in the endometrium, or during the process of miscarriage. The timing of the research was considered when the immunity of the patient was normalized after the last pregnancy failure. It may explain why our results are in opposition to the theory of potential immunological mechanisms of RPL: graft rejection-like alloimmunity, innate immune system hyperactivation (e.g., the hyperactivity of CD56 cells) and organ-specific auto-immunity dependent on autoantibodies [1,2,8].

### The Influence of Sildenafil Citrate on PBMC Cells

Our previous study demonstrated that RPL patients treated with sildenafil suppositories presented decreased peripheral blood NK activity tested in the luteal phase of the menstrual cycle [14]. While SC is used in the therapy of RPL, the data on its influence on maternal immune tolerance are scant [48,50,51,52,53]. Therefore, we assessed the influence of the drug on the production of cytokines and the percentage of regulatory T cells, which may influence NK cell activity. We found that sildenafil managed to improve TGF-β production in the PBMC of fertile women. TGF-β has a strong anti-inflammatory effect and together with IL-10 or IL-15, primarily regulates and shapes the uterine NK cells [32].

Additionally, SC profoundly decreased the concentration of the main pro-inflammatory cytokines: IL-6 and IL-12 in the PBMC cultures of healthy women. Karakhanova et al. performed in-depth analyses of the immunomodulatory function of SC on mice and obtained similar data concerning IL-6 [25]. Results obtained by Luigi et al. showed that sildenafil was able to decrease IL-6 concentration in human fetal cardiomyocyte (Hfcm) cell cultures after TNF-α and IFN-γ stimulation [54].

IL-12 is the most important cytokine in Th1 cell formation and proliferation, which drives NK cell activation with the enhanced secretion of cytotoxic cytokines and the ability to kill tumor cells directly [32]. Thus, a decrease in IL-12 and the elevation of TGF-β as well as IL-10 may improve the effect of SC on NK cell activity and other inflammatory cells.

Nunes et al. reported strong anti-inflammatory properties of sildenafil in the demyelination animal model [55] through the reduced secretion of IL-2, TNF-α, INF-γ and IL-1β and the improvement of IL-10 production [56]. We obtained similar results considering IL-10 cytokine only. IL-10 is a crucial cytokine for trophoblast survival [1] and a key contributor to the balance of pro- versus anti-inflammatory signals that orchestrate proper pregnancy outcomes [57]. Therefore, SC might exert positive effects on pregnancy outcomes through the improvement of IL-10 production in the case of RPL women.

Contrary to the findings of El-Far et al., [48,55,58] our research showed that sildenafil increased the production of TNF-α in the PBMC of RPL patients and had no influence on NKT cell percentage in RPL patients [48].

Recent results confirmed our previously published observations, where SC upregulated the concentration of TNF-α in the serum collected from RPL patients after therapy with SC intravaginal suppositories [59]. Kaleta et al. achieved analogous results in the PBMC cultures of healthy men [60]. However, it was reported that increased NO level after sildenafil treatment might result in NF-κB activation and TNF-α release from the immune cells [14]. Notably, IL-10 and TNF-α share the extracellular signal-regulated kinases (ERK proteins) in the activation pathway [61], which may explain the phenomenon of a simultaneous increase in IL-10 and TNF-α production observed in our research. A high level of TNF-α during pregnancy is considered detrimental. However, the inflammatory process caused by TNF-α is crucial for trophoblast invasion, and the development of spiral arteries over the first stages of pregnancy. Thus, the effect of SC on TNF-α concentration in vitro found in the present study might be positive in vivo during pregnancy by supporting trophoblast implantation and angiogenesis in cases of patients with an exhausted immune response [62,63,64,65].

## 5. Conclusions

Remarkably, we observed a lower concentration of pro-inflammatory cytokines in RPL patients PBMC compared to fertile women PBMC cultures, which might suggest the exhaustion of the immune system in RPL women. Our study is the first to report the influence of sildenafil citrate, a PDE5 inhibitor, on a wide spectrum of human cytokine production in vitro, and confirm that SC exerts anti-inflammatory and immunomodulatory effects on human lymphocytes.

We found that SC enhanced the production of TNF-α and IL-10 by the PBMC of RPL patients. Therefore, sildenafil might modulate the endometrial environment and enhance the decidualization, implantation process and angiogenesis. The elevated concentration of IL-10 in the RPL group and the impaired production of IL-12 and IL-6 in healthy female PBMCs cultured with sildenafil might partially explain diminished peripheral NK cells activity demonstrated in our previous research, and might be utilized in the treatment of RPL women with an imbalanced Th1 response.

## Figures and Tables

**Table 1:** Characteristics of study and control group.

	RPL Patients, *n* = 22	Control Group, *n* = 21
Age (years)	36.70 ± 4.48	37.40 ± 1.90
Number of miscarriages	3.66 ± 1.57	0
PCOS	11	0
MTHFR variant (C677T or A1298C)	18	0
Mean level of homocysteine	12.48 ± 1.63 µmol/L	-

**Table 2:** The percentage of CD4^+^CD25^+^FOXP3^+^ −Treg, CD4^+^CD25^+^IL-17^+^-activated Th17 and CD4^+^CD25^+^FOXP3^+^IL-17^+^(Th17/Treg) positive cells determined among CD4^+^CD25^+^ of the cultured PBMC of fertile women and RPL patients with and without SC for 48 h, results presented as the mean and SD in case of the normal distribution of data, and median and IQR1-IQR3 in case of the non-normal distribution of data, (CG—control group, *n* = 20; RPL—study group, *n* = 20; SC—sildenafil citrate).

Percentage of Positive Cells (%), Median, IQR1–IQR3 or Mean ±SD	Fertile Women PBMC (CG)	Fertile Women -PBMC (CG) + 400 ng/mL SC	*p* Value CG vs. CG SC	RPL PBMC	RPL PBMC + 400 ng/mL SC	*p* Value RPL vs. RPL SC	*p* Value CG vs. RPL
CD4^+^CD25^+^	2.2 (1.6–2.9)	2.0 (1.4–2.8)	ns	2.4 (1.8–3.2)	2.3 (1.7–3.4)	ns	ns
CD4^+^CD25^+^FOXP3^+^	23.1 ± 14.8	21.7 ± 14.5	ns	31 ± 21.3	29.3 ± 20	ns	0.157
CD4^+^CD25^+^IL-17^+^	3.3 (1.25–7.0)	3.2 (1.3–9.5)	ns	3.85 (1.95–8.9)	3.6 (2.3–8.7)	ns	ns
CD4^+^CD25^+^FOXP3^+^IL-17^+^	0.6 (0.3–0.9)	0.7 (0.4–1.2)	ns	0.7 (0.4–2.0)	1.3 (0.6–3.7)	0.018	ns

**Table 3:** Differences in the percentage of CD3^+^CD56^+^CD44^+^CD161^+^ cells among CD3^+^CD56^+^ cells in the PBMC cultures of healthy women and RPL patients, presented as the mean and SD, in case of the normal distribution of data mean and ± SD, or in case of the non-normal distribution of data, median and IQR1-IQR3 (CG—control group, *n* = 20; RPL—study group, *n* = 22; SC—sildenafil citrate).

Percentage of Positive Cells (%), Median, IQR1–IQR3 or Mean ± SD	Fertile Women PBMC (CG)	Fertile Women -PBMC (CG) + 400 ng/mL SC	*p* Value CG vs. CG SC	RPL PBMC	RPL PBMC + 400 ng/mL SC	*p* Value RPL vs. RPL SC	*p* Value CG vs. RPL
CD3^+^CD56^+^	8.4 (4.8–16.0)	6.9 (3.8–16.5)	ns	6.1 (3.3–10.5)	6.0 (3.6–13.2)	ns	ns
CD3^+^CD56^+^CD44^+^CD161^+^	20.4 ± 8.9	20.2 ± 8.0	ns	24.1 (16.8–34.2)	26.7 (17.7–32.8)	ns	0.073

## Data Availability

The data presented in this study are available on request from the corresponding author.

## Supplementary Materials

The following are available online at https://www.mdpi.com/article/10.3390/jcm10143115/s1, Figure S1. Gating strategy for CD3CD56CD44CD161 NKT cells, Figure S2. Gating strategy for CD4^+^CD25^+^Foxp3^+^ and CD4^+^CD25^+^IL-17^+^ lymphocytes enumeration. Figure S3. Correlations between the concentration of pro-inflammatory cytokines in culture supernatants before and after supplementation with 400 ng/mL of SC, spearman’s rank correlation coefficients, Figure S4. Pearson’s “r” factor correlations between inflammatory cytokines where the concentration of the cytokine was similar in CG and RPL group before and after supplementation with SC. Table S1. The concentration (pg/mL) of selected cytokines in the culture supernatants of the PBMC cells of fertile and idiopathic RPL women cultured with and without 400 ng/mL sildenafil citrate.

## Abbreviations

APA	antiphospholipid antibodies
APS	antiphospholipid antibody syndrome
cGMP	cyclic guanosine monophosphate
CTLA–4	cytotoxic T cell antigen 4
GMP	guanosine monophosphate
IL	interleukin
IR	insulin resistance
MTHFR	methylenetetrahydrofolate reductase
NKT	Natural Killer T cell
NO	nitric oxide
NOS	nitric oxide synthase
PBS	phosphate-buffered saline
PCOS	polycystic ovary syndrome
PDE-Is	phosphodiesterase inhibitors
PDE	phosphodiesterase
RPL	recurrent pregnancy loss
SC	sildenafil citrate
TGF-β	transforming growth factor β
TNF-α	tumor necrosis factor α
Treg	T regulatory cell
