# Supplementary material for: Decreased Production of TNF-α and IL-6 Inflammatory Cytokines in Non-Pregnant Idiopathic RPL Women Immunomodulatory Effect of Sildenafil Citrate on the Cellular Response of Idiopathic RPL Women
Source: J Clin Med. 2021 Jul 15;10(14):3115. doi: 10.3390/jcm10143115 (PMC8303721; doi:10.3390/jcm10143115)
Supplement: Supplementary file 1 [file jcm-10-03115-s001.zip › jcm-1235700-supplementary.pdf]

Decreased production of TNF- α and IL-6 inflammatory cytokines in non – pregnant idiopathic RPL women. (Immunomodulatory effect of sildenafil citrate on the cellular response of idiopathic RPL women).

Monika Kniotek[1], Michał Zych[1], Aleksander Roszczyk[1], Monika Szafarowska[2], Małgorzata Jerzak[2]

[1]Department of Clinical Immunology, Transplantation Institute, Medical University of Warsaw, Nowogrodzka 59, Warsaw, 02-006, Mazovian Voivodeship, Poland

[2]Department of Gynecology and Oncological Gynecology, Military Institute of Health Sciences, Szaserów 128, Warsaw, 04-141, Mazovian Voivodeship, Poland

a.

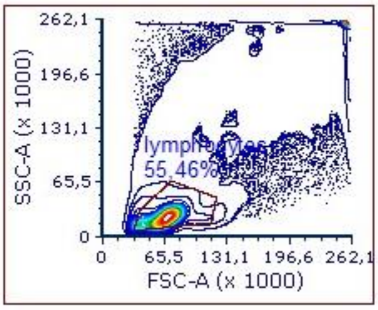

b.

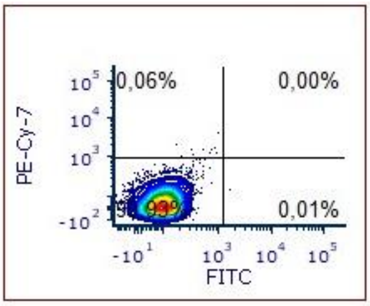

c.

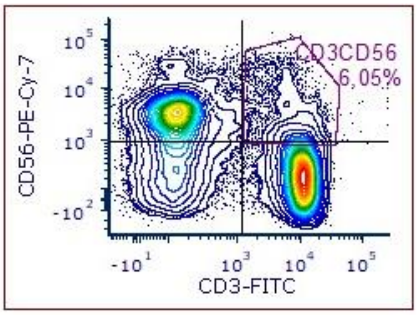

d

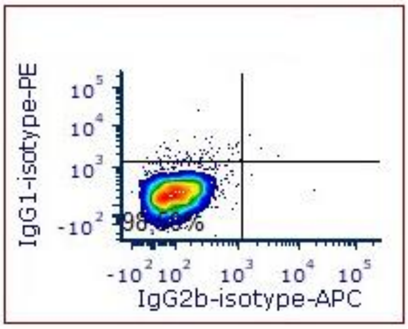

e

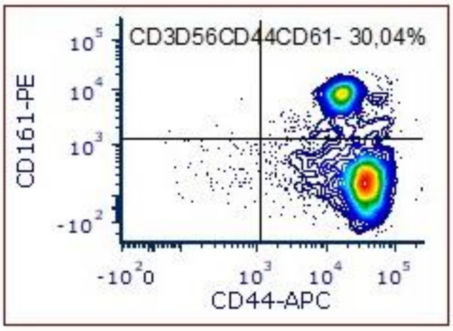

Figure S1. Gating strategy for CD3CD56CD44CD161 NKT cells. a) a region based on forward light scatter (FSC) versus side scatter (SSC) was drawn around the major lymphocyte population, b) unstained cells serve as fluorescence control for CD3CD56 positive cells, c) CD3CD56-positive cells among lymphocytes were identified as Pe-Cy-7 (fluorescence above 10^3 decades on the y-axis) and CD56-FITC positive cells (fluorescence above 10^3 decades on the x-axis), d) isotype controls for CD161-PE and CD44-APC antibodies were used to set the boundaries of specific fluorescence, e) among gated $CD3^+CD56^+$ cells CD161-PE (the red fluorescence above $50x10^3$ decade) and CD44-APC cells (fluorescence above 10^3 decades for APC) were identified.

a. b. c.

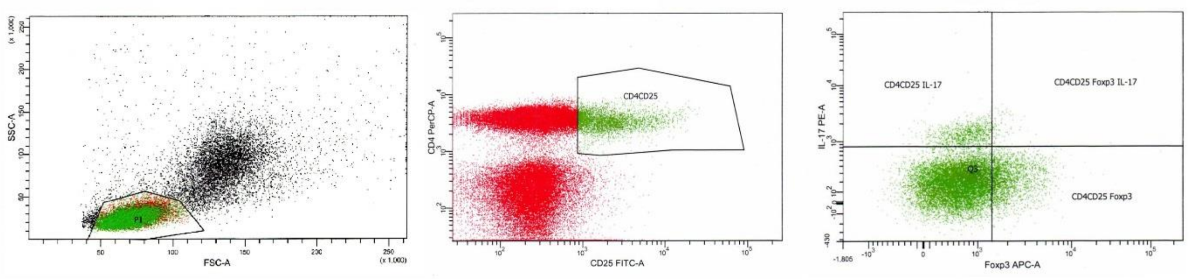

Figure S2. Gating strategy for CD4+CD25+Foxp3+ and CD4+CD25+IL-17+ lymphocytes enumeration, a) lymphocytes were identified using FSC/SSC dot plot, b) CD4CD25-positive cells among lymphocytes were identified as PerCP (above 10^3 decade on y axis) and CD25-FITC positive cells (fluorescence above 10^3 decade on x axis) (green gate), c) then among gated CD4+CD25+ cells IL-17-PE (the red fluorescence above 10^3 decade) and Foxp3-APC cells (fluorescence above $1x10^3$ decade for APC) were identified.

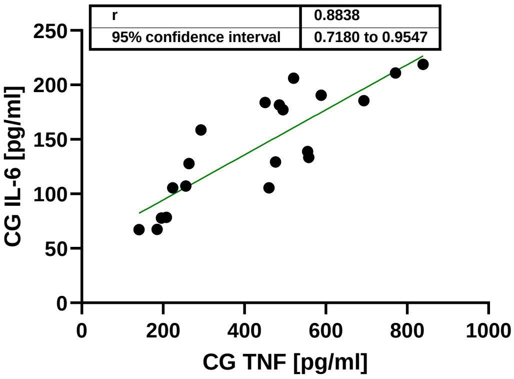

a.

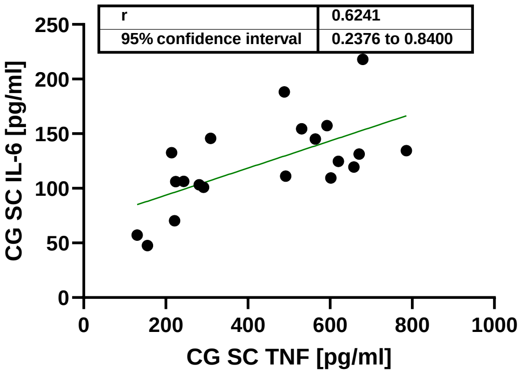

b.

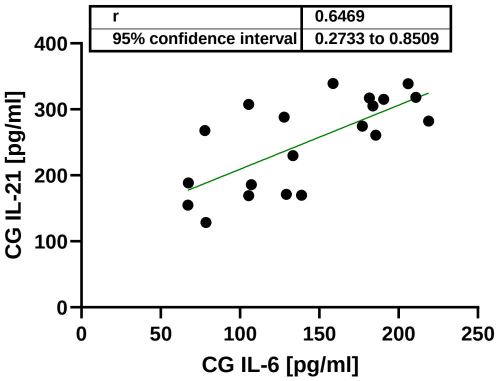

c.

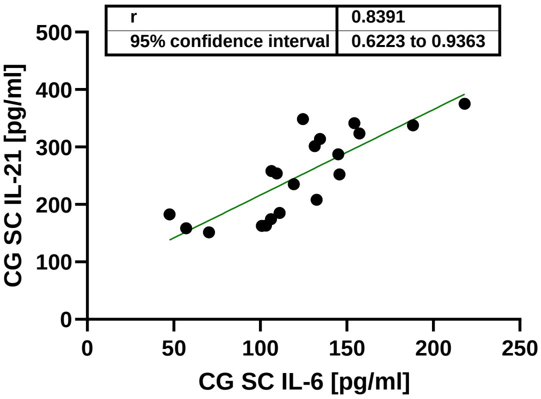

d.

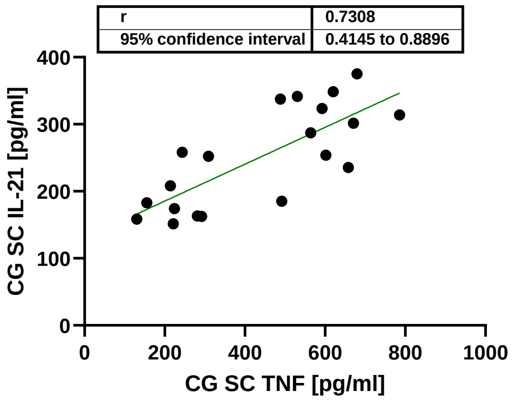

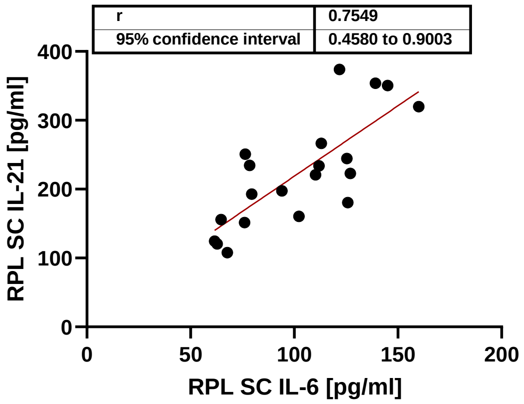

e. f.

Figure S3. Correlations between the concentration of pro-inflammatory cytokines in culture supernatants before and after supplementation with 400 ng/mL of SC, spearman's rank correlation coefficients. green or red line - "R_s "Spearman correlation coefficient, a -e - control group, f - RPL patients, CG-control group, RPL – recurrent pregnancy loss patients, SC – 400ng/ml of sildenafil citrate.

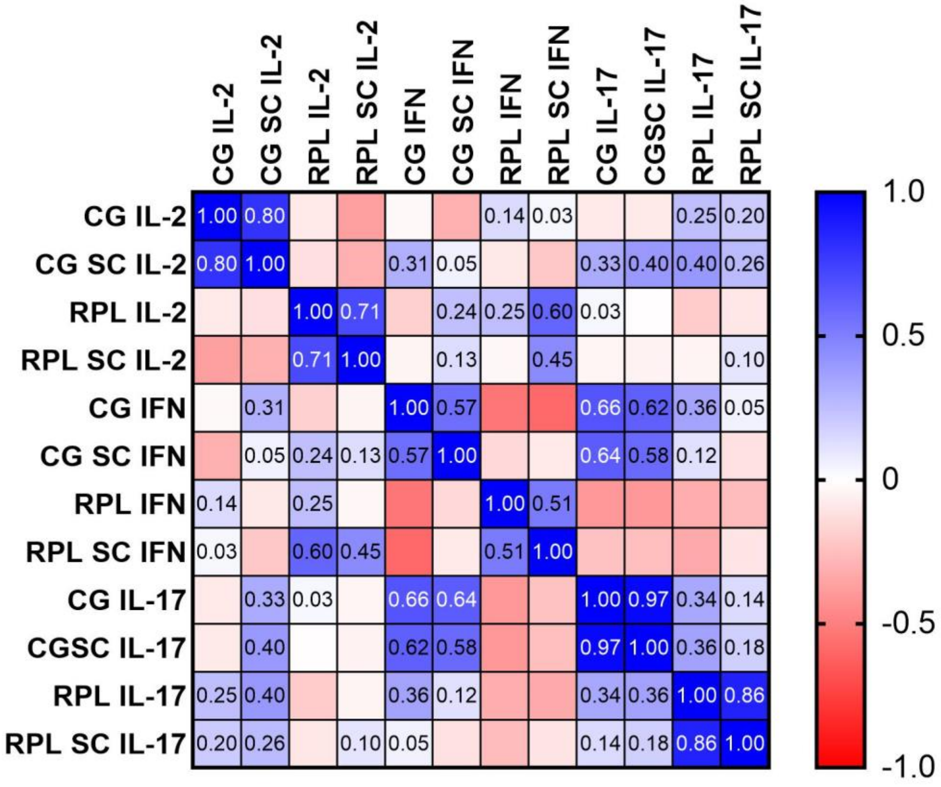

Figure S4. Pearson's "r" factor correlations between inflammatory cytokines where the concentration of the cytokine was similar in CG and RPL group before and after supplementation with SC. The blue colors stand for a positive correlation – r > 0, red colors for a negative correlation – r < 0, (CG – control group, RPL – idiopathic recurrent pregnancy loss patients, SC – sildenafil citrate).

Table S1. The concentration (pg/mL) of selected cytokines in the culture supernatants of the PBMC cells of fertile and idiopathic RPL women cultured with and without 400 ng/mL sildenafil citrate. Data presented as mean and ±SD in case

of the normal distribution of data, or median and IQR1-IQR3 in case of the non-normal distribution of data (CG – control group, n=20; RPL – study group, n=21; SC – sildenafil citrate).

**Concentration of cytokines (pg/ml)**	**Fertile women PBMC (CG)**	**Fertile women - PBMC (CG) + 400 ng/ml SC**	**p value CG vs. CG SC**	**RPL PBMC**	**RPL PBMC + 400ng/ml SC**	**p value RPL vs. RPL SC**	***p* value CG vs. RPL**
TNF- α	468.3 (247.6-556.0)	490.2 (238.5 - 606.5)	ns	231.0 (210.9-308.3)	254.0 (211.4-307.1)	0.026	0.006
IL-6	136.6±58.7	132.8±48.0	0.002	97.35 (71.5-128.3)	106.3 (78.0 - 125.5)	ns	0.007
IL-12p40	8.1±1.59	7.4±1.61	0.010	8.11 ±2.55	7.2±2.5	ns	ns
IL-21	271.1 (182.1-309.3)	253.1 (180.6-316.3)	ns	246.2±92.62	223.2±79.21	0.078	ns
INF-γ	198.9 (166.5-260.1)	184.7 (157.2-244.3)	ns	176.1 (154.3-211.4)	181.6 (159.8-199.08)	ns	ns
IL-2	102.8±29.39	104.8±27.45	ns	111.6±25.34	110.2 (95.0-114.2)	ns	ns
IL-17	222.6±72.1	221,0±79,4	ns	196,6±67,80	200.9 (187.4-255.4)	ns	ns
IL-4	395±104.10	385±105.51	0.078	368.8±105.35	360.7±110.91	ns	ns
IL-10	1316 (1250.0-1444.5)	1283.5 (743.8-1684.1)	ns	1297.1±361.8	1526.0±491.7	0.019	ns
TGF-β	355.0±156.6	424.1±120.8	0.04	472.7±130.5	467.3±121.8	ns	0.02
